# Medico Legal Society of Chicago, Dr. Lydston’s Paper

**Published:** 1896-09

**Authors:** 


### Society Notes.

### Medico-Legal Society of Chicago.

Discussion on papers read January 12, 1896, the subject being "Crime and Criminals." Dr. G. Frank Lydston said:

*Mr. President and Gentlemen:*

The last symposium of the Medico-Legal Society of Chicago was a memorable one, and reflected great credit upon our organization, both in the character of the subjects presented and the manner in which they were considered.

Less than fifteen years ago, the writer ventured to publish an essay on the pathogeny of vice and crime, with a consideration of the methods of correction and prevention, which was very warmly received—so warmly that the resultant "roasting" is still a pleasant recollection.

Some years ago the writer also presented a contribution to the subject of sexual perversion, in which an attempt was made at the classification of perverts and the medico-legal importance of the subject suggested. Others had broken the ice in this direction, but the paper was nevertheless received quite as warmly as the previous paper upon the crime question.

But things seems to be different nowadays. It is apparently safe at the present time to discuss the subjects mentioned from a materialistic standpoint without losing caste in the medical profession. It is even safe to call a spade a spade, before this particular society at least.

Verily, "the world do move," and I congratulate the members of this society upon the attempt which it has made to help the cause along.

I have introduced the subject in this manner because I am convinced that my experience has signified something of importance—namely, that the efforts of materialistic students of the vice and crime question have been in the right direction and supported by scientific facts.

So popular has the cause become within recent years that we are menaced with the usual danger in such a cause—over enthusiasm and the misdirected zeal of dilettante and sensationalists. Especially is this true in the direction of expert testimony involving the question under consideration.

Our last symposium furnished material enough for several meetings, and I feel myself inadequate to the task of discussing even a small proportion of the questions which are set forth, but I have taken the liberty of presenting, in a somewhat disjointed fashion, some of the points which impressed me as being of especial importance:

Dr. Hamilton Wey's* paper was a very important and suggestive one, but it seemed to me that he neglected an opportunity to contribute to the truly scientific aspect of the question by laying undue stress upon its clinical features. I would also call attention to the fact that many of the cases which Dr. Wey so forcibly presented are by no means a type of the sexual pervert—they comprise a special and limited class; sexual perverts from vicious surroundings and example, associated with sexual deprivation. Such subjects, perhaps, might be appropriately called "pseudo-perverts," as contrasted with the subjects of congenitally faulty sexual differentiation, or certain cases in which

*Dr. Wey is surgeon of the Elmira Reformatory, N. Y.

the subject is congenitally fairly sound, but in whom the sexual equilibrium is very easily disturbed, with resulting physical sexual abberation by various unnatural impressions received at, or about, the age of puberty.

Dr. Wey, quoting from Krafft-Ebing, says, "Sexual perversion is especially frequent among civilized races, this being explained by the frequency of abuse of the sexual organs, and by an abnormal constitution of the nervous system."

This statement requires some modification: Sexual perverts are very frequent among some savage tribes. History says that it was frequent among the ancient Scythians. It is found among certain nomadic tribes of Mongolians. Among the Pueblo Indians and some other tribes of our American Aborigines it is very prevalent. The *mujerado*, or male prostitute, of the Pueblos is a very important factor in their primitive social system.

There is no question in my mind but that sexual perversion is more frequent, and more varied in its forms, in civilized society than among primitive social systems,—indeed, it seems to me probable that were it not for one very important etiological factor, savage races would escape perversion altogether—I allude to reversion of type.

This factor, practically ignored by Krafft-Ebing, is, it seems to me, one of the powerful causes of sexual perversion. One who grants the truth of the evolutionary doctrine, and takes the trouble to observe the habits of the lower animals, should not fail to understand this particular point.

Dr. Wey states that the morals of the country are not superior to those of the city in respect to sexual perversion. I beg leave to differ, on purely physiological as well as clinical grounds. The environment, habits, and healthy constitution of the denizen of the country, are all conducive to a higher grade of morality than the average city standard. Perverted sexuality is rarely fed by good, rich blood, or fostered by out-of-door exercise.

Occasional physical and moral degenerates are found, but the average standard is high. Were it not for the constant influx of healthy blood from the country, our large cities might perhaps in time become populated largely with perverts, criminals and idiots.

Dr. Wey's examples are the refuse of country life—the exceptional cases. It is not fair to take the criminal country boy as the criterion of the normal status of his district. Nor is the criminal boy of the city a fair standard for judging the frequency of sexual perversion. Look higher up the scale—there's the place to get statistics on this point. I repeat, Dr. Wey's cases are isolated instances, and no fair criterion of the moral status of the communities from which they came.

In case this assertion should be questioned, let it be remembered that neither masturbation, rape, nor fornication, constitute phases of sexual perversion. Many *dilettante* students of the subject confuse sexual abberrations and sexual crimes of all kinds with sexual perversion. Sexual perversion is a disease, and, as already stated, most prevalent in more advanced states of civilization. It is often associated with the highest type of intellectual and social refinements. Civilization of a high order develops extreme sensitiveness of nervous equipoise, and it is by no means surprising that sexual perversion should be so frequent.

Dr. Wey is correct in his assertion that resorts for sexual perverts exist in our midst. They not only exist, but are known to the police. To any curious or skeptical listener, I will venture to say that it would be an easy matter to secure the services of a police official who will show from twenty to fifty male perverts in a single evening right here in Chicago.

We will carry this matter a little further. There are fashionable resorts in this city in which no females are allowed to become inmates unless they are willing to cater to the depravity of the perverts who patronize them. Let it be noted that the patrons of these places are men of fashion and excellent social standing—men of the upper crust of society. It is a notorious fact that the largest, most gorgeous and most popular house of ill fame in this city is run with an eye to the patronage of this very class of men—who are more perverted than the outcasts they patronize!

And yet we boast of our civilization!

These fashionable men are less excusable than the criminal pervert—less excusable than the congenital pervert—for their perversion is the result of satiation. That of the incarcerated criminal is often due to vicious example, sexual necessity and the lack of diversion;—that of the congenital pervert is a matter over which he has little or no control. Can we say the same of the educated and would-be refined men whom I have mentioned?

[Here followed a classification of sexual perverts, which we omit.—Ed.]

In passing, I desire to call attention to the fact that psychic differentiation of the sexes is never perfect until after the period of puberty. Mutual masturbation or any abnormal sexual impression at this period may result in acquired sexual perversion. Boarding schools are a prolific source of this disease. Parents can not be too careful in preventing close intimacies between growing children. Strange as it may seem, attention was first emphatically called to the importance of this subject by a French novelist. Mdlle. Giraud constitutes very instructive reading upon this question as applied to the female.

I agree with Dr. Wey as to the advisability of castration of certain masturbatory lunatics, but I am none the less convinced that the sexual aberration in these cases is but a complication, not a direct cause. I do not believe that masturbation alone ever causes insanity. In every case I believe that physical degeneracy—usually brain disease—is at the bottom of the mental derangement.

The boy of normal brain and will, can not be made insane by masturbation. If this were not true, there wouldn't be sane people enough left to care for the army of lunatics which would soon over run us.

Castration for true sexual perversion is not likely to do much good in a curative way. Such patients—to use a metaphor—were, in many cases, castrated before they were born, at least as far as the normal sexual desire and receptivity are concerned. Passive perverts are especially likely to be resistant to this method of cure.

In some cases there is nothing to remove which is likely to impress the patient's sexual constitution to any extent. Such patients are often cryptorchids, or have atrophied and useless testes. In some cases castration would perhaps be quite likely do no more than make the active party to perverted sexuality become a passive factor in such acts.

So much for the cure of sexual perversion by castration:

In spite of what has been said, I unhesitatingly assert that every sexual pervert, male or female, in whom all measures of cure have failed, should be castrated. As a cure? No. As a punishment? A thousand times no!—But to protect the race. A large proportion of sexual perverts are capable of procreation. To illegitimate procreation there is no bar,—to legitimate procreation, the bar provided by our highly intelligent social system is $1.50—a matter easily overcome.

Certain acquired cases can be cured, the congenital varieties rarely if ever. They are hardly fit to live, not at all fit to procreate. We may not kill them unless they should happen to commit murder and haven't money enough to hire a lawyer to defend them,—so let us castrate them and prevent them doing further mischief.

Dr. Daniel's communication to the society [A Plea for Reform in Criminal Jurisprudence], was an excellent and forcible presentation of some of the most important points in the field of criminology. There are some points that appear to demand especial consideration.

One of the most important points brought out by Dr. Daniel is the treatment of the sexual criminal, with especial reference to the negro rapist in the South. Some years ago, I presented an exhausted discussion of this subject to this society. To this paper Dr. Daniel did not allude, because, as he has since informed me by letter, he had given it extended mention in his paper before the Medico-Legal Congress in 1893.* In that paper he called attention to the futility, both of lynching and legal executions or imprisonment. Nowhere in the history of civilization has the futility and barbarity of capital punishment been so well shown as in the punishment of negro rapists in the South.

The negroes who perform the acts under consideration are the lowest and most ignorant of the race. They cannot read the newspapers, and it is conceivable that a negro may be hung, or burnt at the stake, without the negroes of the adjoining county becoming apprised of it. The lower class negro is subject to attacks of *furore sexualis* which completely remove any inhibitory impressions which he may have received, even though, in his rational moments, he knows that swift and terrible vengence will be meted out to him for the crime of rape. He is usually a religious fanatic, who sees the gates of heaven yawning wide open for him just beyond the scaffold.

Those gates are ever hungry for the fruit of the gallows tree,

---

*Dr. Lydston is mistaken. A careful reading of my paper will fail to find any reference to sexual perverts—that subject having been discussed in my paper of August, 1893, before the Medico-Legal Congress.—Ed.

and your negro fanatic needs no priest or clergyman to bid him *bon voyage*.

The Zulu crops out in his not very remote descendant, on such occasions. Death is no punishment to such as he, and its moral effect is but transitory on those about him. What the rapist needs is an ever present object lesson, and one which puts him beyond the power of committing further criminal acts of a like nature.

A negro clergyman of education, in commenting on my paper on this subject, said: "The conceded superiority of the white race has much to do with rapes committed on white women by negroes. Art, literature and religion, combiné to inflame the passion of the negro for white women. Your faries, nymphs, goddesses and angels are all white. Did you ever hear of a black angel? The result is an inflamed passion and an exaggerated curiosity on the part of the negro."

It is my opinion that a few castrated negroes scattered throughout the South, would do more good than a multitude of executions. The colored clergyman whom I mentioned, suggested that the offender's ears should be cropped, so that he might be suitably branded externally. I accept the amendment most cordially.

Dr. Daniel becomes a trifle sentimental over the case of a boy of 17 years of age, who was committed for killing a policeman—"his first killing." This boy behaved well for seven years, and Dr. Daniel says that he was "cured."* Cured of what? He never *was* a professional murderer. His was a sporadic case of criminality, and one in which it is impossible to say that the same conditions would not lead to another murder.

No matter how well behaved he may have been, that boy was still dangerous. From an extensive experience I am free to say that convicts doing time for murder are nearly always well behaved, because, 1st: their crimes were committed in most cases under exceptional excitement. 2d. Good behavior affords the only hope of liberty of men who are imprisoned for a definite term, but who may at sometime excite the pardoning sentiment in somebody or other.

Beware of all well behaved murderers unless there be something more than good behavior as an evidence of cure. Re-

---

*Mistake: See article referred to. It reads He was "reformed"—the word put in quotations.

pentance is of course no evidence—they are nearly all repentant.

Understand, I believe that quite a large proportion of murderers might be liberated without danger; but I simply desire to call attention to the difficulty of deciding this point. Your sporadic murderer may be "cured" until such time as certain sources of excitement control him. He is much more difficult of analysis than the habitual criminal.

Dr. Daniel suggests a commission of medical men to diagnose the grades of criminality and decide upon appropriate measures of treatment.

Now this is all very well in theory, but will it work? Who shall form the board, how shall they be appointed, and upon what absolute data shall they form their diagnoses? I have been an enthusiastic student of criminology for many years, but such a board is far beyond my hopes, however much I may desire such a consummation of modern criminal anthropology.

I do not think it will be necessary for me to expatiate upon the difficulty of getting accurate and honest opinions on the questions involved in the physical diagnosis of crime. Those who would like a practical demonstration of this point would do well to review the expert evidence in several of our recent criminal cases. If one would seek for monumental evidence let him review the Prendergast case. We may imagine the workings of a board composed of expert, experts, newspaper experts, experts for revenue only, experts for glory alone, and experts in everything but their own moral obliquity!

I firmly believe that the existence of such a board for six months would, with the meagre data at our command, bury the science of criminal anthropology so deep that it could never be resurrected! Let us make haste slowly. Let us rather bear those laws we have, than fly to boards we know not of, that would make confusion worse confounded.

With such a board in operation, it is conceivable that the smooth, clever, polished, handsome assassin with good physique and well formed head, might live in clover, while his poor degenerate brother would be compelled to eat "salt horse" and corn dodgers.

No, a board for the diagnosis of crime would be a dangerous thing. Let us rather concern ourselves with the conditions that produce the criminal, and seek for the best means for their correction.

It has seemed to me that we are working at a disadvantage by considering the criminal of to-day as being the most important factor in the crime problem. Dr. Oliver Wendell Holmes hit the nail on the head when he said, "If you want to reform a man, begin with his grandfather." A little more money spent in manual and military training schools for our young boys and girls, would do more good than ten times the amount spent in punishment. A little less correction, much less preaching and more care of children by the State is what is necessary.

I wonder if any of our good people ever realize that most of the disease and crime of modern society creeps in through the marriage license window?

A license to breed criminals, insane, epileptics, syphilitics, consumptives, drunkards and paupers costs $1.50. The matrimonial concern having been licensed, is blessed by the church, or its queer copartner in the wrong—the justice shop, and then the output of the factory of degenerates thus started is regulated entirely by the sweet will and physical capacity of the partners in the concern.

Guard the marriage licence window conscientiously, and the doctors and criminal lawyers will have little to do bye and bye, but to condole with one another, or congratulate themselves that midwifery, real estate and corporation litigation still survive.

In the consideration of so vital a question as the management of the criminal class it is a pity that sentiment should dominate so largely as has been the case from time immemorial. The sentimentalist and his natural ally, the preacher, have joined hands on the question, and to them the world has looked for the reformation for which it has waited in vain. Such practical treatment as the question has received, has been chiefly in the direction of devising ways and means to punish the criminal, the building of penal institutions and scaffolds, with the expensive law machinery which leads thereto. Society says to the criminal, "We will punish you thus and so, if you commit such and such crimes." And then society sets about devising ways and means to save the elect from its own laws, and has split hairs to such an exceeding degree of fineness, that there lies between the thieving corporation or the absconding millionaire, and the petit larceny fellow who steals to live, an impassable gulf—one at least, across which mammon alone can build a bridge.

Society makes crime, manufactures its own criminals, and winks at the violation of its own laws in high places. It gives the criminal all facilities—the best of inducements for carrying on his avocation, and then threatens to punish him if he follows the path cut out for him. Above all, society gives the criminal a chance to breed. Crime seems to be more profitable and comfortable, on the average, than honest labor.

What have our preachers, moralists, sentimentalists and law makers accomplished? They have spent the energy and money of the people for nothing. Every penal institution, every expensive process of criminal law, is a monument to the stupidity and wastefulness of society—an expenditure of money and energy to *cure* a disease which might be largely *prevented*. The arraignment of society by Buff Higgins, as he stood upon the gallows, was the most truthful and forceful presentation of the crime question that was ever delivered.

We have millions for foreign missions, millions for sectarian universities, millions for courts of law and penal institutions, but nothing for the salvation of those children of to-day who will be the criminals of the future.

The first and worst injury that society inflicts upon the criminal is, *allowing him to be born*. The criminal has a good and just cause against us—his revenge is sometimes as logical as it is sure. [Alas: poor Elliott!—Ed.]

Natural as sentiment may be in certain quarters, it is remarkable that a practitioner of law, and a man of the highest standing in his profession, should come before this enlightened body and protest, upon sentimental grounds, against what would inevitably be a great advance step in sociological progress.

I do not oppose sentiment in general if it be healthy; but sentiment applied to sociological problems is—well it *should* be a back number. It certainly is misplaced. If I am not in error, sentiment is a powerful sledge for driving an idea of the justice of a doubtful cause into the addled pates of a dozen of those people described to the criminal—often aptly—as his "peers," but I doubt its effectiveness in this presence.

If sentiment were logic; if oratory were argument, if eloquence were reasoning, my friend, Mr. Elliott, would have settled the question of the castration of criminals for all time. But is it settled? I wot not my friends.

With all due respect to Mr. Elliott, and while acknowledging the power of his eloquence, I would respectfully remark that while his plea for his client was a model one, he—well, he didn't pick the right kind of a jury.

The salient points of Mr. Elliott's Niagara of eloquence, as far as I was able to gather them, were these, viz.:

*Firstly.* He apparently doesn't favor the castration of criminals. (Reasons not clearly shown, but largely a matter of personal sentiment.)

*Secondly.* "The maximum limit of virility in the male sex is fifty to sixty years." (He failed to cite his authority for this particular statute of limitations.)

*Thirdly.* "The hens lay eggs in Kansas." (A remarkable combination of ornithological and geographical knowledge which shows how simply abstruse problems may be presented.)

Now, as to the first of these points, no one can disagree. In behalf of a down-trodden sex, however, I resent the second proposition—as applied to doctors—anyhow, and if current rumors be correct, "there are others." As for the third proposition, my innate sense of gallantry impels me to accept, without qualification, the gentleman's assertion in regard to the oviparous productiveness of the western pullet, whatever her breed may be—although, like most "lay" observations, it is at least open to argument.

As sentiment was hurled at us so liberally by my distinguished friend in his eloquent and touching plea in behalf of the criminal testicle, suppose we give him a dose of his own medicine:—

How would the castration of a criminal compare with that recent execution in St. Louis, in which a poor devil was hanged, and the rope breaking, he was strung up again after undergoing some forty minutes of torture while the old noose was being removed and a new one adjusted? There are many like instances. How would castration compare with some of our bungling electrocutions?

How would the operation compare with the execution of Sherry and Connolly some years ago, in which case, while it was shown that one of the men committed a murder, it was impossible to show which man was guilty. On general principles, both were hanged;—it is hardly necessary to state that both were poor and friendless.

Capital punishment—the scaffold, has been a failure; it is brutal, barbaric, and a relic of the dark ages, yet my friend

Elliott has practiced all around it—he has led some criminals gently to it, and has led others who happened to be on the right side of his eloquence, away from it. Sentiment is an unstable quality and an indefinite quantity, it seems.

One case of bungling execution, one execution of an innocent person, is enough to damn the entire system of capital punishment for a thousand years! Does any rational man deny that such cases have happened? Mr. Elliott quotes the Scriptures in his affecting plea for the testicle. Strange how those same Scriptures can be used by some people on both sides of the fence! For instance, the Bible says, "an eye for an eye, a tooth for a tooth," and the law says, "Moses was all right, *ergo* we will execute." And then, when we threaten the criminal testicle, along comes Mr. Elliott and begins to hedge, saying, "Oh, that doesn't fit this case; the Bible says, "Multiply and replenish the earth."

If Mr. Elliott can utilize those India rubber Scriptures can not we do the same? Does not the good book say somewhere, "If thine eye offend thee, pluck it out." How will that fit the case of society vs. the criminal testicle?

We will admit the validity of the "multiply and replenish the earth" proposition, for the sake of argument. Criminals were scarce in those days, and when the Lord told man to "multiply his kind," He forgot to qualify as to *what* kind. Even though the Lord told man to multiply his kind with due consideration for the future criminal, should we allow the latter to go on multiplying? We haven't followed the Scriptures literally in other directions, why in this?

Then, too, in the old Bible days, was it not fashionable to take the malefactor outside the city gates and *stone* him?—what better precedent does the court demand?

There is one feature of castration which makes it far superior to capital punishment in most cases. Executions do not punish, and are but an evanescent lesson to others.

An old darkey was sent to report the hanging of a negro for murder, and commented on the execution as follows: "While de man wuzer standin' dar, Marse Sherriff done out an' read er big long paper to 'im. Marse John, whaffo' dey want ter read dat paper fo'? Dey'se gwine ter kill dat man; he won't know nuffin' 'bout dat termorrer." Could a better argument against the fallacy of capital punishment be advanced than that of the poor old negro?

A few castrated murderers, habitual criminals and rapists scattered throughout the community, would be most efficient aids to the criminal memory. But it is not as punishmen tthat castration is chiefly recommended—it is as a preventive of crime. It is *the* rational method of protecting society against its criminal excreta. Nor should it be restricted to the criminal clases alone. Some incurable lunatics, like criminals, would best be cared for in a similar manner. Shall we go on, allowing the unfit to breed and burden society with their progeny, or shall we take some rational means of protection?

I have quoted Oliver Wendell Holmes as saying, "If you want to reform a man, begin with his grandfather." I offer an amendment: "If you want to reform the criminal, castrate *both* his grandfather and grandmother."

There is but one substitute: Take the children of to-day who will be the grandfathers of future generations of criminals and make useful citizens of them. And yet, this failing, castration again comes into play.

Mr. Elliott spoke of the viciousness and savagery of the eunuch of the east as an illustration of the dangers of castration. He then forgot his life preserver, and got in over his neck. In the first place, before he makes any deductions from the characteristics of the eunuch, he must compare him with the race from which he sprang. I presume that castrating the "Ahkoond of Swat" would not have produced a nineteenth century dude. The Oriental eunuch comes from a race of savages. Those female counterparts of the eunuch, the Amazones of Dahomey, are not only savages, but after being made practically neuters, are trained by savages for savage deeds.

Another point: They are castrated young and trained afterwards; while we are advocating the castration of the adult criminal. The result will not be the development of savage instincts in the criminal, but if the experience of countless generations goes for anything, the operation will be likely to tone him down to a marked degree.

That Mr. Elliott is absolutely wrong is shown by observation of animals and by thousands of cases of castration in the human subjects. Did the choir boys of Rome develop blood thirsty instincts? If so, their soprano voices were a misfit; and the holy, humane church castrated them to no good purpose.

There is not a practicing physician who does not know of dozens of women who have been asexualized for the relief of ovarian disease, and yet where, oh where is our Amazonian army? Pursuing its usual course of peaceful Sunday church going and Monday shopping on State street,—there has been no increase of the mortality rate among the dry goods clerks and no swelling of the divorce calendar by hen-pecked husbands.

In conclusion, I desire to say that the advocates of castration demand it, not for all criminals, but for habitual and incurable types,—for rapists and possibly for some murderers. As far as the latter are concerned, their execution is a waste of raw material.

Let them have the privilege of choosing between scientific experimentation under anæsthesia, and castration. They well might expiate their crimes by benefiting scientific medicine. As for capital punishment—away with it.

And now, my legal friends, follow the example of modern medicine, and do more in the prevention of the diseases you treat. Help us in our labor of benefitting humanity at our own expense.

